# EI competencies as a related but different characteristic than intelligence

**DOI:** 10.3389/fpsyg.2015.00072

**Published:** 2015-02-10

**Authors:** Richard E. Boyatzis, Joan M. Batista-Foguet, Xavier Fernández-i-Marín, Margarida Truninger

**Affiliations:** ^1^Department of Organizational Behavior, Case Western Reserve University, ClevelandOH, USA; ^2^ESADE Business School, BarcelonaSpain

**Keywords:** emotional intelligence, cognitive ability, emotional intelligence competency, social intelligence competency, cognitive competency

## Abstract

Amid the swarm of debate about emotional intelligence (EI) among academics are claims that cognitive intelligence, or general mental ability (*g*), is a stronger predictor of life and work outcomes as well as the counter claims that EI is their strongest predictor. Nested within the tempest in a teapot are scientific questions as to what the relationship is between *g* and EI. Using a behavioral approach to EI, we examined the relationship of a parametric measure of *g* as the person’s GMAT scores and collected observations from others who live and work with the person as to the frequency of his or her EI behavior, as well as the person’s self-assessment. The results show that EI, as seen by others, is slightly related to *g*, especially for males with assessment from professional relations. Further, we found that cognitive competencies are more strongly related to GMAT than EI competencies. For observations from personal relationships or self-assessment, there is no relationship between EI and GMAT. Observations from professional relations reveal a positive relationship between cognitive competencies and GMAT and EI and GMAT for males, but a negative relationship between EI and GMAT for females.

## INTRODUCTION

General cognitive ability (*g*) has been consistently shown to predict job performance in many studies and meta-analyses over the decades (Nisbett et al., 2012). But in the last 10–15 years, emotional intelligence (EI) has also been shown to predict job performance in an increasing number of studies (Fernández-Berrocal and Extremera, 2006; Joseph and Newman, 2010; O’Boyle et al., 2011; Joseph et al., 2014). A debate has emerged as to whether these two individual characteristics are the same, different, or complimentary. A meta-analysis of published papers as of 2009 claimed that *g* showed more predictive ability of job performance than EI (Joseph and Newman, 2010), although both were significant. In some recent studies EI has been shown to have greater predictive ability than *g* (Côté and Miners, 2006; Boyatzis et al., 2012). This study is an attempt to examine the relationship between a behavioral approach to EI and *g* and help create a more comprehensive perspective on these characteristics and the implications for future research.

A major criticism of the EI concept was found in Matthews et al. (2002), but they confused theoretical distinctions and measurement issues. More recently, Webb et al. (2013) said, “Although there is general agreement that the ultimate relevance of EI lies in its ability to predict important life outcomes (e.g., quality of interpersonal relationships, academic or occupational success), debate persists in how best to operationalize…and measure EI…” (p. 154). The debate is confusing at times because EI itself has been conceptualized and measured in various ways.

In some approaches, EI is viewed as the ability to be aware of and manage one’s emotions and those of others which have been called stream 1 and stream 2 measures (Ashkanasy and Daus, 2005; O’Boyle et al., 2011). For example, Mayer et al. (1999) see their concept of ability EI as a formal type of intelligence specialized in the field of emotions and thus related to *g*. Initially, while they had no intention to relate EI to job and life outcomes, later studies have shown ability EI to associate with performance but not as strongly as other approaches (O’Boyle et al., 2011; Miao et al., unpublished). Another perspective sees EI as a set of self-perceptions, which are different from but related to personality traits (Bar-On, 1997) more than *g*. Although this approach along with some measures known as Trait EI (Petrides and Furnham, 2001) have been shown to predict job performance (O’Boyle et al., 2011), they also show a consistently strong relationship to personality traits (Webb et al., 2013). Regardless, it has been filed under the uninformative label of “mixed models” (Mayer et al., 1999).

Another way to understand EI involves observing behavioral manifestations of EI, in terms of how a person acts, as seen by others (Boyatzis, 2009; Cherniss, 2010; Cherniss and Boyatzis, 2013). Known as behavioral EI, it offers a closer link to job and life outcomes. Notably, it has been shown to predict job performance above and beyond *g* and personality (Boyatzis and Goleman, 2007). Nonetheless, this approach has been clustered incorrectly with self-perception approaches and filed under the same label of mixed models (Mayer et al., 1999), also called stream 3 (Ashkanasy and Daus, 2005; O’Boyle et al., 2011).

Although many issues emerging from these varied studies and meta-analyses call for further research, in this paper, we focus on examining the relationship between behavioral EI and *g*, and assessing the potential moderator effects of gender and type of observer or rater.

### BEHAVIORAL EI

Because all of the papers in this special issue of Frontiers in Psychology are devoted to EI and *g*, we will forego an in-depth review of the literature on EI, and instead focus directly on behavioral EI. As mentioned above, EI competencies can be viewed as the behavioral level of EI (Boyatzis, 2009; Cherniss, 2010; Cherniss and Boyatzis, 2013). Competencies have been derived inductively from studies of human performance in many occupations and in many countries (Boyatzis, 2009). Because the identification of a competency and its refinement emerges from performance based criterion sampling, they are expected to be closely related to job and life outcomes. As a result, the EI competencies were discovered and measured as behaviors which were later clustered around intent and became each competency (Boyatzis, 2009).

In Boyatzis and Goleman (2007), EI includes two factors, EI and social intelligence (SI) competencies. EI includes competencies called emotional self-awareness, emotional self control, adaptability, achievement orientation, and positive outlook. In their model, SI includes: empathy, organizational awareness, influence, inspirational leadership, conflict management, coach and mentor, and teamwork. For this paper, we are treating EI and SI competencies as a single construct of EI. When universities wish to use this EI model for student development and/or outcome assessment, two cognitive competencies which have a history of predicting effective leadership, management and professional performance are added. They are: systems thinking and pattern recognition (Boyatzis, 2009).

Behavioral EI as seen and measured through others’ assessment (as compared to self-assessment) shows a consistent prediction or relationship to job and life outcomes (Boyatzis, 1982, 2006; McClelland, 1998; Nel, 2001; Cavallo and Brienza, 2002; Dulewicz et al., 2003; Law et al., 2004; Sy et al., 2006; Dreyfus, 2008; Hopkins and Bilimoria, 2008; Koman and Wolff, 2008; Williams, 2008; Boyatzis and Ratti, 2009; Ramo et al., 2009; Ryan et al., 2009, 2012; Young and Dulewicz, 2009; Boyatzis et al., 2011, 2012; Aliaga Araujo and Taylor, 2012; Gutierrez et al., 2012; Sharma, 2012; Amdurer et al., 2013; Victoroff and Boyatzis, 2013; Mahon et al., 2014; Badri, unpublished). Boyatzis et al. (2012) showed behavioral EI predicted job performance with significant unique variance, controlling for *g* and personality.

According to the dominant classification in Ashkanasy and Daus (2005), there are three different streams of EI research. Salovey and Mayer’s Ability EI as measured by the MSCEIT is stream 1. Although it has shown relationships with school (Brackett et al., 2004), job and life outcomes (Mayer et al., 2008), these were not of primary consideration in its development (Mayer et al., 1999). Whereas ability EI shows no relationship to personality measures, it has shown consistent prediction of *g*, even when controlling for personality (Webb et al., 2013).

Self-perceptions and peer-report measures based on the Ability EI model are clustered within stream 2 (Ashkanasy and Daus, 2005). These measures such as the Trait EI Questionnaire (TEIQue; Petrides and Furnham, 2000, 2001), show similar validity patterns to the MSCEIT but are not as strongly related to *g*, nor job and life outcomes, yet they do show a significant relationship to personality (Webb et al., 2013).

Meanwhile, stream 3 (Ashkanasy and Daus, 2005) clusters both those EI measures based on self-perception and others’ behavioral assessments (i.e., 360°, coded behavior from audiotape or videotape work samples or simulations). Consequently, there is a partition in results within this stream: some measures such as the ESCI (Boyatzis and Goleman, 2007) show a strong relationship and unique variance to life and job outcomes beyond *g* and personality (Byrne et al., 2007; Downey et al., 2011), while others such as the EQ-i (Bar-On, 1997) show a consistent relationship in predicting personality (Joseph and Newman, 2010; O’Boyle et al., 2011). We therefore, claim that clustering self-perception and coded or other perception measures confuses these relationships.

Instead, we support Fernández-Berrocal and Extremera’s (2006, p. 8) comprehensive view of the EI field by which all “approaches try to discover the emotional components that underlie *emotionally intelligent* people and the mechanisms and processes that set off the use of these abilities in our everyday life” (emphasis added). In the authors’ review of the first 15 years of EI research, behavioral EI as seen by others in 360° assessments is considered separately from self-perception approaches focused on moods and internal states, as well as personality traits such as Bar-On’s (1997, 2007; Fernández-Berrocal and Extremera, 2006). Therefore, Boyatzis (2009) extends the work of Fernández-Berrocal and Extremera (2006) to propose an organization of the literature that is framed by the three existing methodological themes: EI ability methods; EI self-perception methods; and EI behavior methods.

In sum, the relationships of EI assessed at any level or with any method are still debated with comparative arguments about its link to *g* and personality. In this paper, we will focus on the relationship between behavioral EI and a measure of *g*.

### GENERAL COGNITIVE ABILITY (*g*) AND INTELLIGENCE

According to Carroll’s (1993) model of intelligence, the various mental abilities are structured hierarchically. General cognitive ability, located at its apex, is “the general efficacy of intellectual processes” (Ackerman et al., 2005, p. 32). Also known as general mental ability, general intelligence, or simply *g*, it is a well-researched construct with a large body of evidence supporting its predictive validity for such important outcomes as job performance and career success (e.g., O’Reilly III and Chatman, 1994; Schmidt and Hunter, 1998; Ferris et al., 2001). As a global ability, *g* can be thought of as the underlying common factor to all types of cognitive processing (i.e., verbal, mathematical, spatial, logical, musical, and emotional). From this perspective, *g* cannot be observed nor measured directly, it must be inferred from the positive correlations among distinct ability measures (Spearman, 1904; Jensen, 1998). As such, *g* subsumes different sets of abilities, each corresponding to a specialization of general intelligence.

General cognitive ability can be assessed through a variety of measures, such as IQ tests (Jensen, 1992; i.e., Ravens Progressive Matrices, Wechsler, Stanford Binet; Nisbett et al., 2012). Similarly, standardized admissions tests have been shown to “fit the general requisites of a measure of general cognitive ability” (O’Reilly III and Chatman, 1994). They also measure verbal and mathematical or quantitative reasoning skills separately. These tests such as the SAT, GRE, GMAT, MCAT, LSAT, and DAT are usually found to have strong correlations with the more direct measures of *g*, (Detterman and Daniel, 1989).

The GMAT is a standardized test that assesses a person’s analytical, writing, quantitative, verbal and reading skills for admission into graduate management programs worldwide. Although the GMAT is not formally validated as a measure of general cognitive ability, it is strongly correlated with the Scholastic Aptitude Test (SAT; e.g., Gottesman and Morey, 2006), which is shown to be a valid measure of *g* (Frey and Detterman, 2004). Considering the structural similarity of these tests (both consist of multiple choice questions that measure verbal and quantitative skills) and the general consensus that the *g*-factor can be measured by obtaining factorial scores across tests of different specific aptitudes, usually verbal and quantitative (O’Reilly III and Chatman, 1994), Hedlund et al. (2006, p. 102) concluded that “like the SAT, the GMAT can be characterized as a traditional measure of intelligence, or a test of general cognitive ability (*g*).” Indeed numerous studies have already used the GMAT as a measure of *g* (e.g., O’Reilly III and Chatman, 1994; Kumari and Corr, 1996; Mueller and Curhan, 2006), the latest of which is a study published in *Intelligence* (Piffer et al., 2014).

We suggest that the EI competencies may show a small, if any relationship to *g*. In fact, correlations between behavioral EI competencies coded from audiotapes of critical incident interviews about work samples and GMAT were not significant (*r* = -0.015, *n* = 200, *p* = ns; Boyatzis et al., 2002). In assessing predictors of sales leadership effectiveness in the financial services industry, Boyatzis et al. (2012) reported that EI as assessed by others showed a non-significant correlation with Ravens Progressive Matrices (*r* = 0.04, *n* = 60, *p* = ns).

In the inductive competency studies, two cognitive competencies repeatedly appeared to differentiate effective performance of managers, executives and professionals (Boyatzis, 1982, 2009; Spencer and Spencer, 1993). They were systems thinking and pattern recognition. The former is defined as seeing phenomenon as a series of causal relationships affecting each other. The latter is defined as perceiving themes or patterns in seemingly random information. As competencies, they are assessed both with a self-assessment and with observations of others as to how often a person demonstrates these behaviors. They are not defined or assessed as an intelligence measure but an indication of how often a person appears to be using these thought processes. As such, we expect them to be related to *g* more than EI competencies even though they are not a measure of *g*.

This leads us to the first two hypotheses for this study:

Hypothesis 1: EI competencies will have a slight relationship to g.

Hypothesis 2: Cognitive competencies will be more related to *g* than EI competencies.

### SELF AND MULTI-RATER ASSESSMENTS

Differences in raters or sources of assessment are likely to play an important role in the findings. Self-perception and multi-rater assessment are different approaches to perceiving and collecting observations of a person’s behavior (Luthans et al., 1988; Church, 1997; Furnham and Stringfield, 1998; Antonioni and Park, 2001; Taylor and Hood, 2010).

Self-assessment measures generally address how individuals respond to questions pertaining to their own emotions, perceptions or thoughts. These measures are easier and faster to administer than others, allowing for low costs of administration (Saris and Gallhofer, 2007). Social desirability is often an issue in self-reported measures (Paulhus and Reid, 1991). That is, respondents may base their answers on a desired state that often leads to inflated views of themselves. The validity of these measures can be improved by including questions that help control for social desirability (e.g., Paulhus and Reid, 1991; Steenkamp et al., 2010).

Used as a stand-alone measure, self-assessment of personality traits, attitudes or behavioral tendencies show acceptable validity (e.g., Furnham et al., 1999; Petrides and Furnham, 2000; Furnham, 2001; Petrides et al., 2006; Bar-On, 2007). Similarly, self-assessed measures of EI show acceptable validity (Bar-On, 1997; Petrides and Furnham, 2000, 2001). However, with regard to EI, self-assessments are also used in combination with others’ ratings. Notably, the difference between self and others’ perceptions is known as the self-other-agreement. This difference is a highly reliable measure of self-awareness (Yammarino and Atwater, 1997).

Multi-rater or multi-source assessments involve different raters from work such as a person’s peers, collaborators, subordinates or bosses, and possibly raters from one’s personal environment. Raters provide observations of a person’s behavior (i.e., what they have *seen* the person do). Research on social cognition reveals that people give more weight to their own thoughts and feelings than to their behavior when forming self-perceptions, but this effect is reversed when forming perceptions of others (Vazire, 2010). Different types of raters may offer unique information about the person being assessed (Borman, 1997). People may behave differently depending on the situation (e.g., at home vs. work; Lawler, 1967).

Other behavioral assessments such as coding from audio or videotapes of critical incidents or simulations may be considered “pure” behavioral measures, but even these measures require people to code them. In the coding, observers are engaged in subjective perceptions and labeling. In such qualitative research, the scholars increase confidence in the data reported by assessing inter-rater reliability. In 360° assessments, greater confidence in the data is developed from a consensual perception of multiple raters. In EI studies, both types of measures attempt to assess how a person has been acting as seen by others (i.e., a behavioral approach to measurement of EI).

A number of studies show that there are differences among boss’s, peers’ and subordinates’ views, and sometimes even others like consultants, customers or clients. Atkins and Wood (2002) claimed specific types of raters were best positioned to observe and evaluate certain types of competencies depending on the personal and working relationships they had with the person being evaluated. For example, subordinates were found to be the best evaluators of competencies such as coaching and developing people, when compared to bosses or peers (Luthans et al., 1988). Similarly Gralewski and Karwowski (2013) showed how, even though teachers are often accurate at assessing the intelligence and academic achievement of their students (Südkamp et al., 2012), they lack the ability to assess less conventional skill areas, such as students’ creativity. Different sources of raters might interpret the same observed behavior in different ways (Tsui and Ohlott, 1988). At the same time each rater source may have idiosyncratic tendencies leading to different observations and measurement error, like errors of leniency, central tendency, and range restriction (Saal et al., 1980). These are likely to be moderated by cultural assumptions (Ng et al., 2012). The research in assessing performance as well as skills and behavior with 360° assessments is summarized in Bracken et al. (2001). Social identity theory would contend that people find more legitimacy in assessing themselves with regard to those of higher status rather than merely more power (Taylor and Hood, 2010), suggesting that raters from work will be more potent than those from home.

Outside of family business, consulting or family therapy, the sources or raters that have been studied do not include family or friends (Bracken et al., 2001), with the exception of Rivera-Cruz (2004). She reported that female managers showed more EI competencies (as seen by others) at home versus work. In a desire to be comprehensive in assessments, data was collected in this study from a wide range of a person’s relations – those from work and from their personal life (Boyatzis, 2009).

With regard to intelligence, it is expected that professional sources (i.e., sources from work) will have more of an opportunity to see and label behavior related to cognitive ability rather than those at home or in one’s personal life.

This leads us to the third hypothesis for this study:

Hypothesis 3: Among personal, professional and self-assessment of a person’s competencies, professional sources will show the strongest relationship of EI and cognitive competencies to g.

### GENDER DIFFERENCES

In self-assessment, an extensive body of literature validated by a recent meta-analysis showed strong evidence of male hubris and female humility: the tendency of males to have inflated views of their abilities, opposite to females’ propensity to under-estimate their worth (Furnham, 2001; Szymanowicz and Furnham, 2011). At the same time, there may be a gender bias in the type of *g* measures themselves as Furnham (2001) proposes that results may be based on the fact that most of these measures are “male normative”. That is, they include specific tasks, such as spatial processing or mathematical reasoning at which males have been shown to do better than females.

As to others’ ratings of EI competencies, stereotyping will likely affect peers perceptions of males versus females, even in the same setting (Taylor and Hood, 2010). Social identity theory, along with social comparison theory and self-categorization theory are expected to result in attributions made to females differently than those made to males even if their behavior was the same (Sturm et al., 2014). For example, Taylor and Hood (2010) reports that even though female MBAs appear to be more assertive and self-confident than other female samples, sexist bias in perception results in males being seen as more assertive and confident than females. However they did find that predicted ratings of others showed a gender difference: “women leaders believed that others would rate them lower than the actual ratings they received” (p. 542).

In light of these findings, we propose females may be subject to sexist discrimination in their multi-source assessments, particularly those from raters at work. This suggests there may be an interaction of both gender and rater in the relationship between EI and *g*.

This leads us to the fourth hypothesis for this study:

Hypothesis 4: Gender moderates the relationship of EI and cognitive competencies to g.

## MATERIALS AND METHODS

Data were collected on 641 part-time and full-time MBA students from 23 countries, in a leading European business school, between 2006 and 2013. 30% were females, with an average age of 33 years for females and 34 years for males. As part of the MBA, the students took a required course called Leadership Assessment and Development which is based on the Intentional Change Theory (Boyatzis, 2008). In the course, students were asked to complete a self and multi-rater assessment of EI competencies. All data were collected under the informed consent an ethical guidelines of ESADE Business School.

### MEASURES

#### Emotional Intelligence Competencies

We used the Emotional and Social Competency Inventory – University Edition (ESCI-U; Boyatzis and Goleman, 2007), a 70-item survey instrument which measures 14 competencies of two types: cognitive and emotional. The first type is composed of two cognitive competencies: systems thinking and pattern recognition. The other, includes 12 EI competencies: emotional self-awareness, emotional self control, adaptability, achievement orientation, positive outlook, empathy, organizational awareness, influence, inspirational leadership, conflict management, coach and mentor, and teamwork. Because the behavioral manifestations of these competencies are frequently observed in a variety of different situations they have been operationalized with as many as five indicators per competency. Psychometric properties of the test based on samples of 62,000 completions of the ESCI and 21,000 of the ESCI-U both reveals each scale shows model fit and satisfies criteria for discriminant and convergent validity (Boyatzis et al., 2014). A wide variety of validation studies on the test were reviewed earlier in this paper and in Wolff (2008).

Competencies can be considered to be the behavioral approach to emotional, social, and cognitive intelligence (Boyatzis, 2009). As such, the student is asked to solicit others from their work and life to complete the test about their behavior. The students had an average of 4.2 others complete the test for each of the 641 subjects in this analysis (standard deviation equals to 1.6). It is believed that multi-source assessment, such as 360°, provides protection against social desirability because of the distinct sources of responses.

Researchers have traditionally placed more emphasis on testing hypotheses on the relationships among constructs than on bridging the gap between abstract theoretical constructs and their measurements (i.e., epistemic relationships; Bagozzi, 1984). In our case, measurement error is particularly dangerous because it affects ESCI as a GMAT predictor leading to biased estimates of the structural effects (Frost and Thompson, 2000). Therefore, before estimating these effects, we examined the ESCI construct validity^1^.

Since we suspected that the ESCI factorial structure provided by the personal and the professional raters could be different as a function of their different perspectives^2^ of the MBA students’ behavior, we have modeled the data separately. Two confirmatory factor analysis (CFA) models have shown that both sets of raters were consistent with the hypothesized 13-factor (i.e., the competencies) model^3^.

For purposes of exploring our research question, we distinguished three types of sources, or assessments in this study. We used a classification provided by each respondent at the time of completing the test. The responses were grouped as either: self, personal, or professional. One is the assessment provided by the student about himself or herself. Another source was personal, such as a spouse/partner, friends, or family members. Professional sources were bosses, peers, subordinates or clients from work or classmates in the MBA program. There were a few cases in which personal or professional assessments were missing, these cases were dropped resulting in a final sample of 624 individuals with personal and 611 with professional assessments available. All had self-assessment.

MBA participants and their raters were asked to indicate the frequency of the behavior on each item on an eleven point-scale ranging from (0) ‘the behavior is never shown’ to (10) ‘the behavior is consistently shown.’ This response set provides higher quality data on this predominantly European MBA population than the usual 5-point scale (Batista-Foguet et al., 2009). The final ESCI-U scores have been mean-centered to ease the interpretation of the parameters in the model. To compute the 360° assessments on the 70 items that constitute the ESCI-U survey, we first obtained for each item, its average score across all professional and personal raters separately, and then averaged across the five items per each competency. This way, our database consisted of 26,264 competency scores from 3 types of raters, on the 12 + 1 emotional, social, and cognitive competencies.

#### General cognitive ability (*g*)

We used the Graduate Management Admission Test (GMAT) as a measure of *g*. For this study we chose to collect our GMAT data from the GMAC, the entity that owns and administers the GMAT, and not through the Admissions Office at the University. We collected the students’ GMAT scores from the first time they took the test. Using GMAT first time scores as compared to the scores with which students were admitted in the MBA program (usually obtained after repeatedly taking the test), enabled a wider range of variation in GMAT with higher dispersion and lower means. We, thus, attempted to minimize the issue of range restriction in GMAT (Oh et al., 2008) and the resulting attenuation bias in the model coefficients. In our sample, the GMAT mean is 602.4, which is a little higher than the overall GMAT for all test takers of 545. The sample’s standard deviation of the GMAT is 79.3, almost two thirds of the reported GMAT deviation (at 121). Therefore, our sample contains individuals with slightly higher GMAT and less “heterogeneous” scores than the population of GMAT applicants.

**FIGURE 1:**
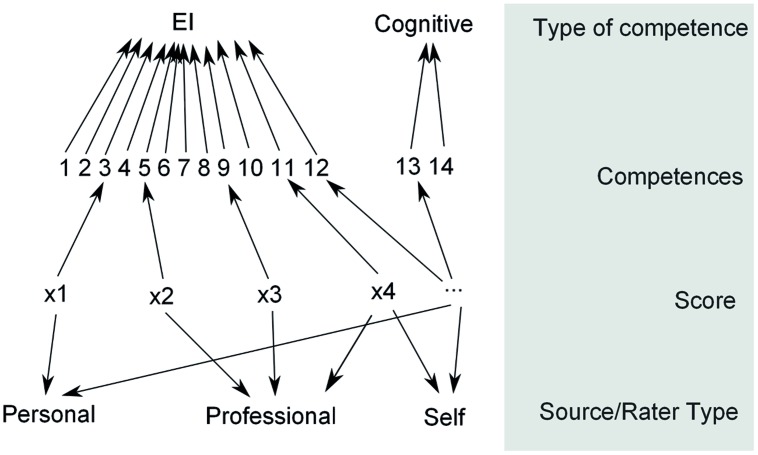
**Emotional and Social Competencies Inventory – University Edition (ESCI-U) data configuration.** The ESCI-U data is framed within two non-nested structures: (1) the raters group, composed of self, personal and professional raters; and (2) the competencies category, withholding 14 competencies, which in turn are sub grouped into two types of competencies: Emotional and Cognitive.

The ESCI-U data are configured in two non-nested structures: (1) the rater groups, varying between self, personal or professional raters; and (2) the competencies category with 13 competencies divided into two types of competencies: cognitive and EI. The hierarchal structure of the data model is shown in **Figure 1**.

The relationship between the ESCI-U and the GMAT scores might be affected by whether the ESCI-U scores on each competency are independent or not from the rater group. Therefore, treating each competency and group of raters as independent might mask important information. To adjust for this possibility, we allowed for a possible dependent relationship between the rater source and the competency category to be freely estimated in our model.

In order to be able to accommodate such a complex data structure and the relationships among the competencies (13 in two groups) and three types of raters, we need a specified model with sufficient flexibility to assign the proper systematic and stochastic variations. A multilevel/hierarchical model with non-nested structures in the first level (raters and competencies) and a nested structure in one of the components (competencies in two groups) is needed.

### BAYESIAN MODEL SPECIFICATION

We chose to analyze the data and test our hypotheses by specifying a Bayesian hierarchical model. The choice to work with a Bayesian model was due to two main factors: (1) the sample was an entire population in and by itself; and (2) it was not a random sample. These issues pose problems in many statistical analyses because traditional frequentist methods are based upon the assumption that the data are created by a repeatable stochastic mechanism. While mainstream statistics treat the observable data as random and the unknown parameters of the population are assumed fixed and unchanging, in the Bayesian view, it is the observed variables that are seen as fixed whereas the unknown parameters are assumed to vary randomly according to a probability distribution. Therefore, in Bayesian models, the parameters of the population are no longer treated as fixed and unchanging as a frequentist approach would assume^4^.

In sum, the main advantages of the Bayesian approach are twofold: (1) it enables highly flexible model specifications (as the one needed to account for the hierarchical structure of our data); and (2) is more appropriate for settings where the data is not a random sample, but the entire population. In addition, it offers a clear and intuitive way to present results. For example, it appears more intuitive by generating *probability* statements about the findings (for more readings on the advantages of Bayesian inference, check the introductory chapters of Gill, 2002; Gelman et al., 2003; Jackman, 2009).

To best accommodate the structure of our data, we used a multilevel or hierarchical model non–nested structure (by competency and rater group). Equation 1 below represents our model specification, which assumes a linear association between GMAT and ESCI-U scores.

**Table:** 

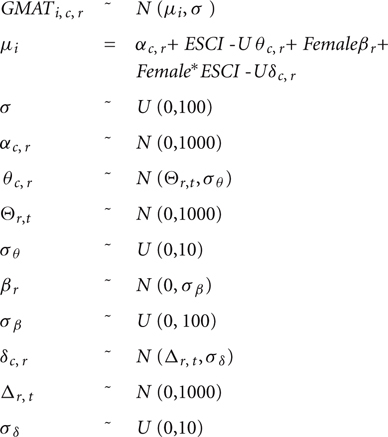

The *i* subscript refers to the individual, the *c* subscript refers to the competency and the *r* subscript refers to the rater group (self, personal or professional). The intercept, α_c,r_, varies by competency and rater group. The parameters that account for the ESCI-U effect, θ_c,r_, have a hyper-parameter^5^, Θ_r,t_, that varies by rater group and by type of competency (i.e., cognitive or emotional).

Additionally, the model includes gender as a source of variation, with coefficient β_r_ varying by group of raters. The moderator effect of gender on the association between ESCI-U and GMAT is also specified, an interaction that is parameterized as *δ_c,r_* – varying by competency category and rater group, with hyper-prior specification that depends on the type of competency.

In total, there are six main parameters of interest to be estimated, which are compared regarding the type of competency (cognitive or emotional) and the rater group. Estimating a model like the one above is not possible using “canned” procedures from mainstream statistical packages. This confounds the other seemingly inappropriate assumptions from frequentist approaches based on maximum likelihood. One technical solution is to use Bayesian simulation techniques, which allow for highly flexible model specifications^6^.

## RESULTS

To test the structure of the 13 competency scales, we used LISREL 8.80 with the covariance matrix to estimate the factorial composition. The same CFA model was specified for professional and personal raters. The fit indexes of the measurement model were satisfactory, as shown in **Table 1**. Factor loadings of the items per competency were above 0.65. The usual global indexes shown in **Table 1** are below or close the appropriate thresholds (Hu and Bentler, 1999). The relatively high values of chi-square were actually due to some irrelevant misspecifications which were magnified due to the high power situation (large sample size and high reliability). We could have released a few constraints on uncorrelated uniqueness but their estimated values would be negligible.

**Table 1:** Confirmatory factor analysis (CFA) model fit for different sources of raters (n = 641).

Raters CFA model	Satorra-Bentler χ^2^ (df)	90% CI RMSEA	*P*-value for test of close fit (RMSEA 0.05)	CFI	SRMR
Professional	4751 (2261)	(0.0404; 0.0437)	1.000	0.992	0.0525
Personal	5399 (2261)	(0.0456; 0.0488)	0.994	0.988	0.0579

In addition, it is well known that these global fit indexes may have limitations resulting in erroneous conclusions (Saris et al., 2009). Therefore, we checked whether: (1) all the estimated values were reasonable and of the expected sign; (2) the correlation residuals suggested the addition of parameters; and (3) the modification indexes and expected parameter changes led to plausible estimates. This process focuses more attention on the detection of misspecification errors rather than solely on the global fit (Saris et al., 2009). It considers the power of the test in addition to the significance levels. The results did not show any significant misspecifications in our CFA model for each set of raters.

Results from a discriminant validity analysis show that all the competencies are adequately discriminated^7^. Discriminant validity was assessed by comparing the square root of the AVE, as shown in **Table 2**, of each reflective construct with the correlations between the constructs, as shown in **Tables 3** and **4**. Despite the relatively high magnitude of some correlations among competencies as shown in **Tables 3** and **4** the results suggested that the 13 competencies were adequately discriminated. To be sure, the two cognitive competencies were integrated into one scale for this analysis. Any model that specified a correlation between two competencies constrained to one has been rejected. Therefore, these results suggested the appropriateness of maintaining the 13 competencies rated by others as separate scales.

**Table 2:** AVE, Cronbach’s α and Omega of the 13 competencies (a) personal and (b) professional (the two cognitive competencies were combined into one factor for this analysis; *n* = 641).

	Constructs	AVE		Cronbach’s α		Ω	
		Pers	Prof				
		(a)	(b)	(a)	(b)	(a)	(b)
[AO]	Achievement orientation	0.519	0.587	0.842	0.875	0.860	0.880
[A]	Adaptability	0.558	0.591	0.856	0.875	0.890	0.910
[CFM]	Conflict management	0.497	0.521	0.824	0.854		
[CM]	Coach and mentor	0.610	0.617	0.882	0.888		
[ESA]	Emotional self-awareness	0.589	0.591	0.874	0.847		
[ESC]	Emotional self-control	0.676	0.731	0.905	0.920		
[E]	Empathy	0.610	0.654	0.885	0.896		
[I]	Influence	0.498	0.534	0.828	0.847	0.840	0.870
[IL]	Inspirational leadership	0.693	0.702	0.913	0.920		
[OA]	Organizational awareness	0.555	0.578	0.852	0.869		
[PO]	Positive outlook	0.652	0.572	0.902	0.868		
[T]	Teamwork	0.654	0.695	0.902	0.914		
[C]	Cognitive	0.543	0.561	0.909	0.916	0.920	0.929

**Table 3:** Correlation matrix of competencies as scored by personal raters (*n* = 641).

		AO	A	CFM	CM	ESA	ESC	E	I	IL	OA	PO	T
**[A]**	**Adaptability**	0.817											
**[CFM]**	**Conflict management**	0.685	0.865										
**[CM]**	**Coach and mentor**	0.626	0.705	0.853									
**[ESA]**	**Emotional self-awareness**	0.560	0.597	0.726	0.749								
**[ESC]**	**Emotional self-control**	0.566	0.720	0.809	0.534	0.460							
**[E]**	**Empathy**	0.588	0.726	0.905	0.814	0.720	0.721						
**[I]**	**Influence**	0.582	0.805	0.802	0.666	0.605	0.500	0.587					
**[IL]**	**Inspirational leadership**	0.724	0.802	0.827	0.786	0.644	0.557	0.596	0.845				
**[OA]**	**Organizational awareness**	0.651	0.870	0.841	0.693	0.568	0.646	0.746	0.783	0.764			
**[PO]**	**Positive outlook**	0.619	0.696	0.670	0.575	0.534	0.553	0.517	0.552	0.734	0.566		
**[T]**	**Teamwork**	0.640	0.780	0.890	0.824	0.594	0.675	0.787	0.653	0.786	0.811	0.674	
**[C]**	**Cognitive**	0.781	0.900	0.793	0.641	0.629	0.632	0.646	0.797	0.769	0.806	0.601	0.646

**Table 4:** Correlation matrix of competencies as scored by professional raters (*n* = 641).

		AO	A	CFM	CM	ESA	ESC	E	I	IL	OA	PO	T
**[A]**	**Adaptability**	0.892											
**[CFM]**	**Conflict management**	0.770	0.840										
**[CM]**	**Coach and mentor**	0.740	0.743	0.875									
**[ESA]**	**Emotional self-awareness**	0.674	0.730	0.799	0.777								
**[ESC]**	**Emotional self-control**	0.509	0.627	0.799	0.593	0.527							
**[E]**	**Empathy**	0.637	0.752	0.930	0.854	0.784	0.788						
**[I]**	**Influence**	0.762	0.853	0.888	0.785	0.803	0.603	0.784					
**[IL]**	**Inspirational leadership**	0.757	0.786	0.793	0.833	0.682	0.538	0.689	0.867				
**[OA]**	**Organizational awareness**	0.686	0.854	0.829	0.738	0.729	0.680	0.825	0.858	0.722			
**[PO]**	**Positive outlook**	0.734	0.742	0.759	0.662	0.603	0.600	0.683	0.705	0.781	0.669		
**[T]**	**Teamwork**	0.683	0.753	0.877	0.903	0.683	0.698	0.887	0.757	0.741	0.830	0.692	
**[C]**	**Cognitive**	0.848	0.908	0.832	0.743	0.776	0.589	0.720	0.869	0.769	0.797	0.652	0.696

With this evidence supporting validity of the scales, we addressed reliability. In **Table 2** we used Cronbach’s α for assessing the internal consistency of each set of five items within each competency. However, for those competencies in which tau-equivalence (Bollen, 1989) was not fulfilled, we used Heise and Bohrnstedt’s (1970) W, which only requires fitting a unidimensional factor analysis model.

Although the two models shown in **Table 2** fulfill the *configural invariance* (same CFA model for personal and professional raters), they showed support for rejecting the condition that the item loadings were the same in both groups of raters (i.e., they had measurement equivalence). Intraclass correlation indexes were not considered because we did not need to aggregate raters into one category of “others.” As a result, the two raters’ perspectives were considered under a hierarchical model specification.

The outcome of a Bayesian model is not a point estimate of the coefficient with an associated standard error, but a complete density distribution of the parameter, which can then be simply summarized by using its median and standard deviation to resemble the traditional frequentist approach of parameter estimates and standard errors. Moreover, percentiles of the parameter’s distribution are used to summarize its credible interval (which is the Bayesian equivalent to a parameter’s confidence interval in classical statistics). In addition, results and substantial interpretations of some of the parameters are presented using graphical figures, in accordance with statisticians’ advice of “turning tables into graphs” (Gelman et al., 2002).

### COGNITIVE VS. EMOTIONAL COMPETENCIES

**FIGURE 2:**
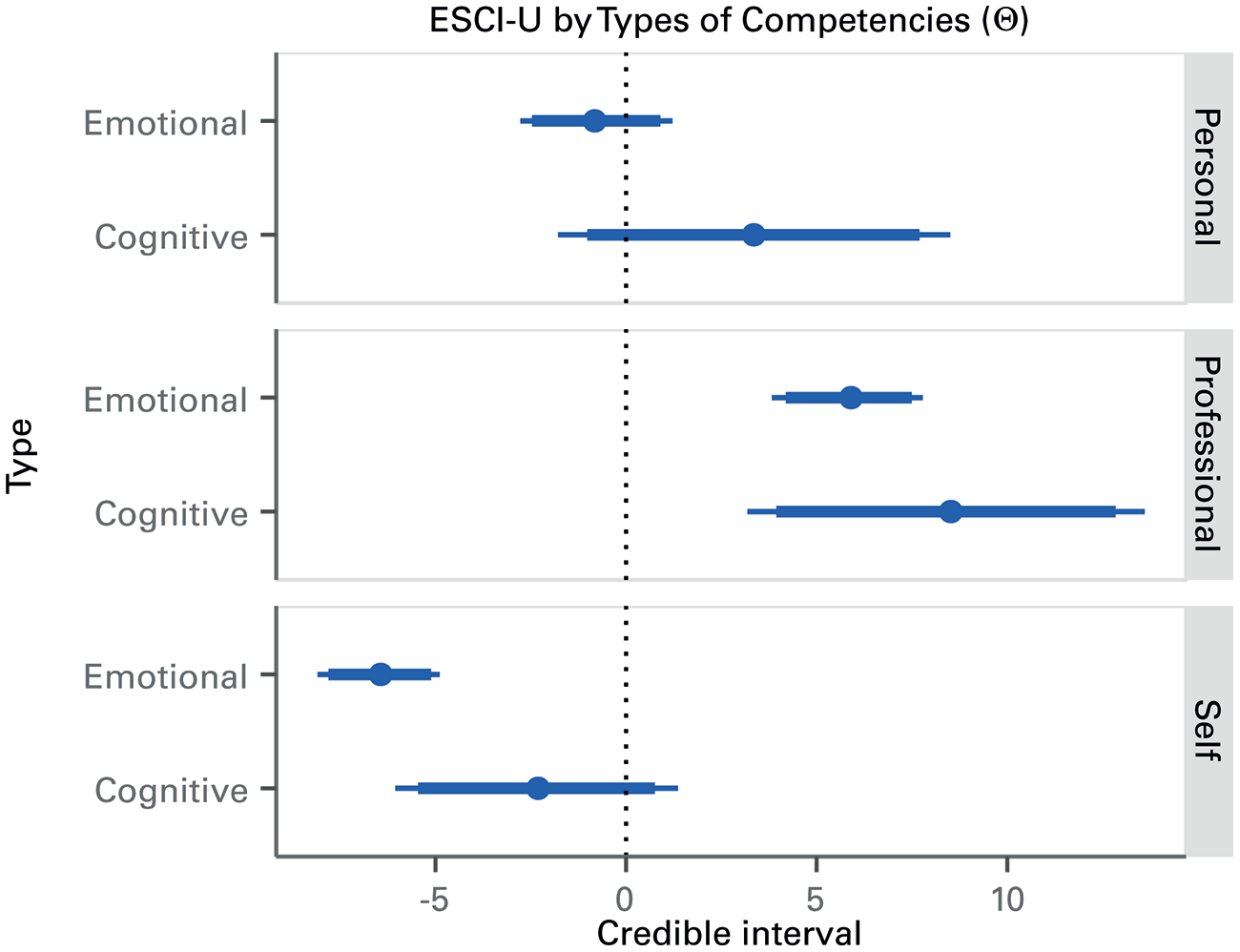
**Caterpillar plot of the posterior distribution of the effects of types of competencies on GMAT scores, by rater.** Credible intervals (median, 90 -thick line- and 95% -thin line-) of the distribution of the Δ parameters that account for the association between the type of competency and the GMAT score. Hence, for the first element (Emotional-Personal), one unit increase in emotional competencies is expected to decrease the GMAT by around one point. However, since the credible interval overlaps zero, there may be weak evidence of an actual decrease.

As mentioned earlier, the main parameters of interest, Θ_r,t_, are those that describe the association between GMAT and ESCI-U competencies depending on which type of competency, cognitive or EI, and which of the three groups of raters are considered. A caterpillar plot is shown in **Figure 2** with the median of the posterior distribution of each parameter and the 90 and 95 percent credible intervals. The parameters can be interpreted as follows: (a) if the distribution crosses the zero point, there is no consistent relationship of significance; and (b) if the line is to the right or the left of the zero point, then it tells us about the relative impact. For example, in **Figure 2**, the cognitive competencies assessed by professional sources have a positive relationship to *g*. The distribution can be said to show that an increase of one unit in the cognitive competencies, as scored by professional raters, is expected to produce an on average increase of around 8.5 units in the GMAT scores. EI and cognitive competencies show no relationship to *g* with observations from personal sources. Observations from professional sources show a positive relationship between EI and *g*. Observations from self-assessment show a negative relationship between EI and *g*. In all three groups of raters the association between GMAT scores and the raters’ evaluation of the cognitive competencies is considerably higher than with the raters’ evaluation of EI competencies. This clearly indicates that GMAT scores are associated in a different way with the ESCI-U scores produced by the three groups of raters. Adding to the main effects mentioned, these results show that the rater group has a moderator effect on the association between ESCI-U and GMAT scores. Therefore we find support for hypothesis 1, strong support for hypothesis 2, and clarity as to the different sources for hypothesis 3.

**Figure 2** also shows that others’ ratings of behavior agree more with each other than they do with self-perceptions. This is a well-established result (Atwater and Yammarino, 1992; Carless et al., 1998) that brings further support to our claim that clustering self-report with others’ ratings or 360° based approaches confuses the relationships of EI to different constructs.

Another way to examine these results is by using probability statements, which is one of the advantages of using Bayesian inference. In this sense, the probability that cognitive competencies are more strongly associated with GMAT scores than the EI competencies ranges between 81.5 percent for professional raters, 92.7 for personal raters and 97.8 for self-evaluations. Therefore, the data offers strong evidence for hypotheses 3.

**FIGURE 3:**
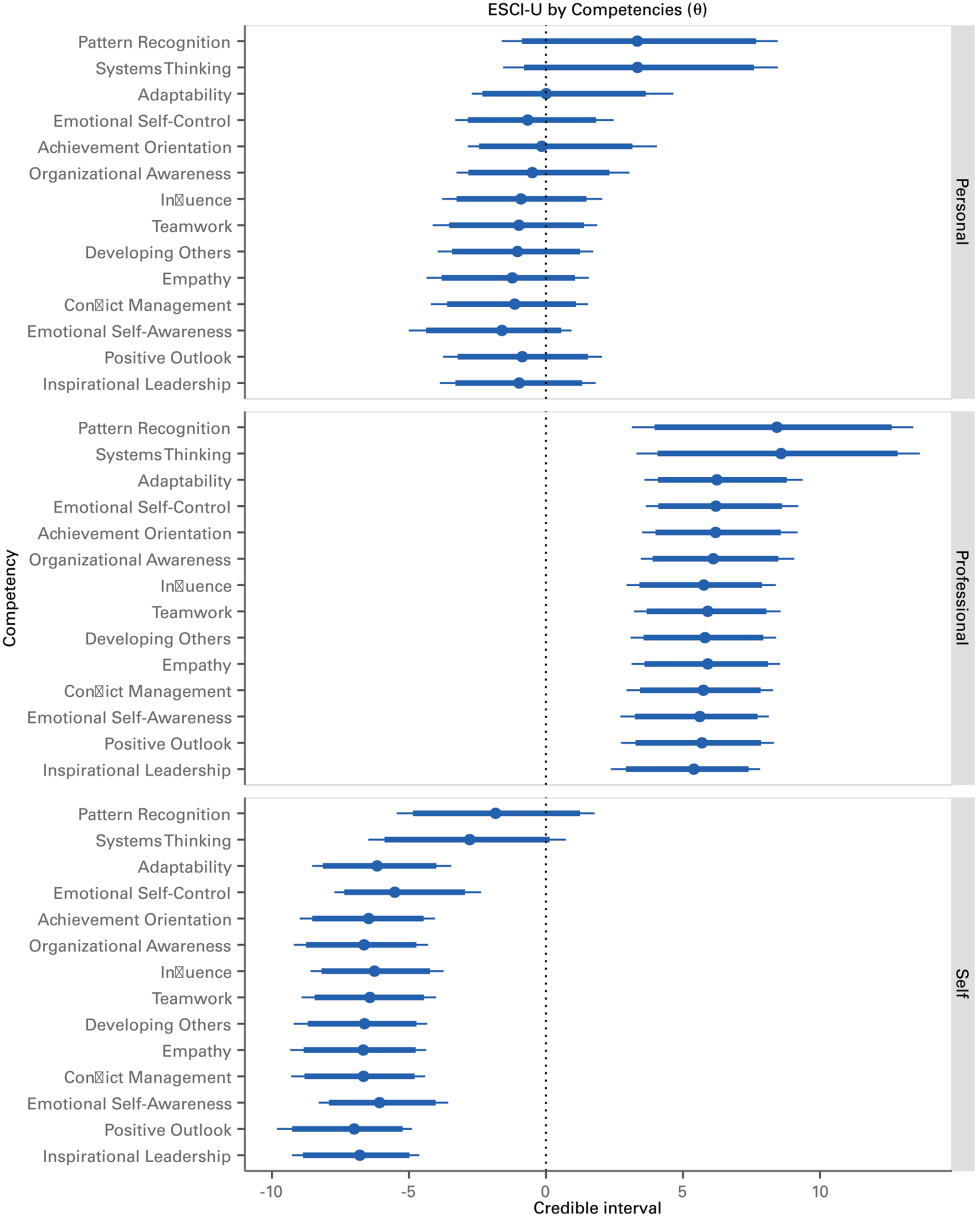
**Caterpillar plot of the posterior distribution of the effects of each competency on GMAT scores, by rater.** Credible intervals (median, 90 – thick line – and 95% – thin line) of the distribution of the *θ* parameters that account for the association between each competency and the GMAT scores.

To provide deeper insight into the consistency of the distributions, **Figure 3** shows the caterpillar plot of all the 52 θ_c,r_ parameters, one per each of the 14 ESCI-U competencies, and the three rater groups. As can be seen, the parameters’ distributions are quite consistent within the EI and cognitive types of competencies results shown in **Figure 2**. The figure can be read as follows, taking as an example the first element of **Figure 3**: an increase of 1 unit in the competency score of pattern recognition by professional raters is expected to generate an on average increase of about 7.5 in the GMAT score. Yet, regardless of which rater perceptions are considered, cognitive competencies always show higher association with GMAT scores than EI competencies.

### THE MODERATOR EFFECT OF GENDER

**FIGURE 4:**
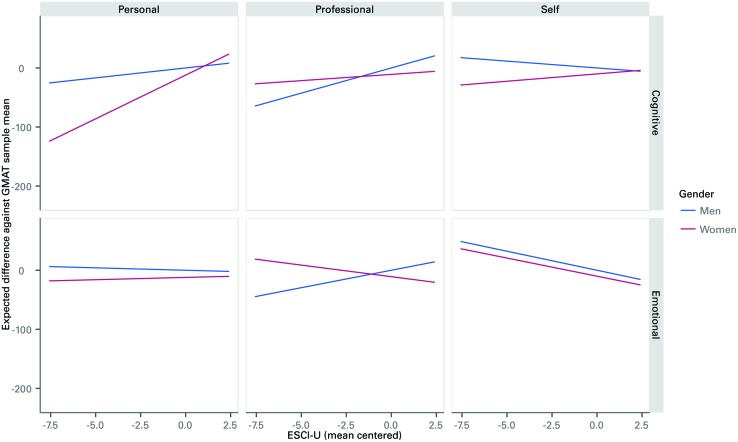
**Expected Moderating Effect of Gender on the Relationship Between ESCI-U score on Cognitive and Emotional Competencies and GMAT, by type of Rater.** The lines represent the expected effect of ESCI-U scores (as departures from the sample mean in the horizontal axis) and GMAT scores (as departures from the sample mean in the vertical axis). Flat lines represent situations in which the association between ESCI-U and GMAT is not clear. Increasing lines can be read as follows: a unit increase in the ESCI-U score for a male/female in an Emotional/Cognitive competency as measured by a specific rater is expected to increase the GMAT score by a certain amount given by the vertical axis.

Regarding the moderator effects of gender, females showed substantially lower associations between EI and *g* than males, as shown in **Figure 4**. In fact, it is negative for observations from each of the self and professional observers and non-significant for personal observers for females. Meanwhile, there is a positive relationship between EI and *g* for males as viewed from professional observers. Although varying in intensity, for all sources for both EI and cognitive competencies, males show a stronger relationship to *g* than females. Regarding cognitive competencies, the relationship to *g* is stronger for males than females from all sources. This provides further support for hypotheses 3 and clarifies why hypothesis 4 is important.

## DISCUSSION

The study examined the relationship between behavioral EI and *g*. We found that cognitive competencies are more strongly related to *g* than EI competencies. EI, as seen by others, is slightly related to *g*, in particular for observations from professional raters for males, but there is no relationship from observations of personal raters, and a slightly negative relationship of EI and *g* from self-assessment. When we examined gender moderating effects, there appears to be a relationship between EI and *g* for males with observations from professional raters. With females, there is no relationship between EI and *g* with observations from personal raters, and a slight negative relationship with observations from professional raters and self-assessment.

In alignment with both Fernández-Berrocal and Extremera (2006) and Boyatzis (2009) frameworks of the research on EI, these results offer further support to distinguish between approaches to EI that are based on self-perception and those that are behavioral. This would add to the literature by supplementing the other approaches and levels of EI with the behavioral approach and helps us develop a more holistic model of the EI. Even with this approach, for males with assessment from professional colleagues, there is a relationship between EI and *g*. It is not as strong as the relationship with cognitive competencies and *g*. But it is there. These findings support the idea reported in other studies that to be effective in management, leadership or professions, we probably need some distribution of EI, cognitive competencies and *g* (Boyatzis, 2006; O’Boyle et al., 2011).

Self-assessment showed a slight negative relationship between EI and *g*. This raises the question as to whether self-perception approaches to EI will be as good in predicting job performance (Taylor and Hood, 2010). But a recent meta-analysis of self-assessment methods did show consistent predictive effects of EI (Joseph et al., 2014). Perhaps for those jobs and professions that involve more analytic activities and tasks which require a higher level of *g* – e.g., a bench scientist, engineering programmer, creative artist or mathematician, self-perceived EI may be relatively less accurate in performance prediction than a behavioral approach.

The gender moderating effects noted may be interpreted as a result of the different expectations and attributions from others to males and females. Whether emerging from stereotyping or social comparison processes, they force what appears to be a more generous attribution of the link between EI and *g* to males than females. One dilemma is that some studies may confound such processes by using a measure of *g* that appears gender biased. For example, the Ravens Progressive Matrices, although considered one of the best measures of *g*, is a visual comparison task (i.e., choosing a figure that fits into a sequence more than others). Since males appear to handle such spatial reasoning more quickly, as a result of prior gender based training and socialization, may give males a different distribution on the results than females. It is recommended that these “male normative” intelligence tests (Furnham, 2001), are paired with the Mill Hill Vocabulary or some such similar test that balances a measure of *g* with specific skills in which females do better than males (Boyatzis et al., 2012).

Overall, the different results from different raters is a reminder that the reality of what you see depends on the direction in which you look, and the color of the lenses you wear.

### IMPLICATIONS

The results suggest that research on EI should examine at more than one level within studies, the ability, trait, self-perception or behavioral levels. It may help in understanding the relevance of EI to life and work outcomes, as well as other constructs in psychology. They also suggest that research on EI should include measures of *g* to show the unique variance contributed by each concept and show the relative power of each. When collecting behavioral EI data, these results suggest that analyses should examine the sources of the observations as a possible moderator or mediator on the dependent variables. For example in this research, it is likely that the professional environment provides more opportunities for the raters to assess *g*-related competencies than the personal environment. It is also crucial to analyze data for gender effects that may not be apparent in more direct, statistical analysis.

Professionals using 360° assessments to coach or develop EI should be prepared to identify systemic differences across gender and rater types. Otherwise, individuals may leave their coaching session thinking they have an actual “problem” with certain raters, when in reality it is a systematic bias shared across the population.

### LIMITATIONS

One of the limitations of this study emerges because the data came from a single school with diverse nationalities. As such, it threatens external validity. The study should be replicated in other schools to insure that a specific school’s selection and admissions criteria have not biased results.

By focusing on MBA students, we also threatened construct validity. Social desirability is one of the most common validity threats associated with the use of questionnaires in this postgraduate population. Raters provided by the individual rated might create a halo effect, an overall positive feeling leading to inflate their perception of how often desirable behaviors are present. Specially, self-assessment is often misguided for this overall positive feeling about oneself, or because being competent is desirable, thus increased positive self-assessment tends to occur. Future research should address this issue as well.

## CONCLUSION

Emotional intelligence exists at multiple levels. The behavioral level of EI shows a different relationship to *g* than other levels or approaches to EI. Different people around us, at home and at work, will see different facets of our behavior, depending on the kind of relationship and rapport they have established. Some raters are best equipped to assess certain competencies than others because they witness frequently the activities that elicit those behaviors. While our study reveals that raters from a professional sphere are more apt to evaluate cognitive competencies, future research would benefit from looking further into discovering which rater type among professionals (boss, colleagues or subordinates) is best suited to assess which ESCI-U competency. The same can be said of the pervasive impact that gender stereotypes and social comparison processes have on observations of others and their interpretations of it. Regarding EI, to be of most help in discovering insights that will be useful to improving our lives, we should be more comprehensive about the variety in approaches to EI and more sensitive to their differences at the same time.

## Conflict of Interest Statement

The authors declare that the research was conducted in the absence of any commercial or financial relationships that could be construed as a potential conflict of interest.

## Footnotes

1 We define validity as “the degree to which evidence and theory support the interpretation of test scores entailed by proposed uses of tests” (American Educational Research Association et al., 1999, p. 9).

2 Since we didn’t assume that Personal and Professional raters have the same perception and aggregate them under the usual “other” category of raters, we have tested their measurement or factorial equivalence (Meredith, 1993).

3 Exploratory Factor Analysis (EFA, Promax rotation) has already shown that systems thinking and pattern recognition competencies correlate on both raters’ perceptions above 0.94. The subsequent confirmatory factor analysis (CFA) didn’t reject the unidimensionality of the 5 + 5 items corresponding to the two competencies, that had ex-ante been assumed as distinct competencies. As a result, in this analysis, we used thirteen instead of the usual 14 factors underlying the ESCI model on this MBA population by having combined the two cognitive competencies into one scale.

4 Instead of a frequentist approach, in this approach a parameter is assigned a prior distribution (based on previous research in the field), which is then updated with the actual data by means of a specified likelihood function, so as to produce a posterior distribution of the parameter (Wagner and Gill, 2005). In fact, in our approach we are not entitled to use a *p*-value (as in frequentist statistics) as the probability of obtaining the observed sample results under the null hypothesis. As mentioned the data is not a sample of a larger population but it is a population.

5 Hyper-parameters provide a clear illustration of the Bayesian view on population parameters. That is, there are no static assumptions made about the mean of a parameter, rather the mean is allowed to fluctuate according to its own probability function. The subscript *r* on the hyper-parameter refers to the gender and the subscript *t* refers to the type of competency, Cognitive or Emotional.

6 As mentioned earlier, Bayesian inference requires researchers to provide prior distributions for the parameters of the model. Given the lack of previous research on this topic, however, the current prior distributions were weakly informative. Consequently, our model has been estimated using Markov Chain Monte Carlo methods, more specifically, the Gibbs sampler. JAGS (Plummer, 2003) has been used for the estimation, while the chains have been analyzed under R with the coda and ggmcmc libraries (Plummer et al., 2006; Fernández-i-Marín, 2013; R Development Core Team, 2013). A total of 5,000 samples of two chains of simulated posteriors have been acquired under different initial values, with a burn-in period of 1,000 iterations. There is no evidence of non-convergence of the series according to the Geweke (1992) test.

7 In addition, as indexes of discriminant and convergent validity (Bagozzi and Yi, 1988), we first checked the average variance extracted (AVE; i.e., the average communalities per competency). As mentioned, the results showed that all items have loadings above 0.65, with competencies having always an AVE above or close to 0.5. In addition, cross-loadings from a previous EFA showed that all the items have much higher loadings with their respective construct (as suggested by Chin, 1998) than with any other competency.
